# A Web-Based Audio Computer-Assisted Self-interview Application With Illustrated Pictures to Administer a Hepatitis B Survey Among a Myanmar-Born Community in Perth, Australia: Development and User Acceptance Study

**DOI:** 10.2196/37358

**Published:** 2023-04-14

**Authors:** Nang Nge Nge Phoo, Alison Reid, Roanna Lobo, Murray Davies, Daniel Vujcich

**Affiliations:** 1 School of Population Health Curtin University Perth Australia; 2 The Viewpoint Organisation Pty Ltd Melbourne Australia

**Keywords:** audio computer-assisted self-interview, ACASI, audio computer, survey mode, application, picture, digital health

## Abstract

**Background:**

Self-administered paper or electronic surveys can create accessibility issues for people with language barriers and limited literacy, whereas face-to-face interviews can create privacy issues and give rise to reporting biases, particularly in the context of sensitive subject matters. An audio computer-assisted self-interview (ACASI) offers an alternative mode of survey administration, and its use has been tested against other survey modes to determine whether the presence of a background narration helps overcome literacy and privacy issues. There are still gaps with the ACASI survey administration because audio narration alone does not assist respondents with limited literacy in choosing response options. To overcome literacy issues, a few studies have used illustrated pictures for a limited number of response options.

**Objective:**

This study aimed to illustrate all the questions and response options in an ACASI application. This research is part of a larger study comparing different modes of survey administration (ACASI, face-to-face interviews, and self-administered paper surveys) to collect data on hepatitis B knowledge, attitudes, and practices among the Myanmar-born community in Perth, Australia. This study describes the 2-phase process of developing a web-based ACASI application using illustrated pictures.

**Methods:**

The first phase was the preparation of the ACASI elements, such as questionnaire, pictures, brief descriptions of response options, and audio files. Each element was pretested on 20 participants from the target population. The second phase involved synchronizing all the elements into the web-based ACASI application and adapting the application features, in particular, autoplay audio and illustrated pictures. The preprototype survey application was tested for user acceptance on 5 participants from the target population, resulting in minor adjustments to the display and arrangement of response options.

**Results:**

After a 12-month development process, the prototype ACASI application with illustrated pictures was fully functional for electronic survey administration and secure data storage and export.

**Conclusions:**

Pretesting each element separately was a useful approach because it saved time to reprogram the application at a later stage. Future studies should also consider the participatory development of pictures and visual design of user interfaces. This picture-assisted ACASI survey administration mode can be further developed and used to collect sensitive information from populations that are usually marginalized because of literacy and language barriers.

## Introduction

### Background

In administering surveys of sensitive topics through face-to-face interview, self-administered paper, and computerized survey modes, research suggests that privacy, limited literacy, and language difficulties were common barriers [1,2]. Audio computer-assisted self-interview (ACASI) is a computerized mode of survey administration in which survey participants can view a questionnaire on a computerized personal device; listen to the background narration that reads the questionnaire aloud; and use regular keyboards, color- or number-coded keyboards, or touchscreen to input their responses. This survey mode has been in use since the 1990s [3-6]. The audio component was added to the computer-assisted self-interview mode to enable self-administration, enhance privacy, and overcome literacy issues [4,5]. Although the narration reads the questions aloud, ACASI still requires some literacy to choose an answer and enter a response [4,5], and studies suggest that this limitation is not fully addressed through the use of color- or symbol-coding [7-9]. ACASI survey participants were also required to understand the language used in the display and audio narration, which could be challenging for some respondents.

The use of pictures in health communication has been shown to be helpful in overcoming literacy issues and language barriers among culturally and linguistically diverse (CaLD) populations [10,11]. A review of the peer-reviewed literature found that the use of photographs, pictures or symbols, and digital technology assisted the comprehension of English or translated text for people from CaLD backgrounds [11]. Another review comparing the use of “text with pictures” to “text-only” in conveying health information concluded that pictures enhanced understanding and facilitated recall, and such impact was more pronounced among people with limited literacy [10].

Although a few studies have examined the use of pictures in web surveys and concluded that such practice could affect survey responses in various manners, for example, as accentuation, interference, and visual context effect [12-17]. Few studies have explored the effects of using pictures in ACASI surveys [18]. With advancements in technology, some ACASI applications and platforms have enabled the use of pictures [19-21]. In a study in India, 4 response options—“yes,” “no,” “do not know,” and “no response”—were illustrated in pictures [22]. In a Malawian study, for a question about the number of sexual partners, the ACASI tool showed images of different numbers of men based on the number entered by respondents [8]. These 2 studies did not report on the impact of the pictures on participants’ ability to understand the question, as intended by the researchers [8,22]. Another study conducted among participants with low literacy in Malawi used a picture booklet with tape-recorded questions as a low-cost alternative to ACASI [23]. They reported that the study participants found it easy to complete the survey using pictures and audio [23].

Despite the positive reports on the use of pictures in overcoming language barriers and literacy issues [10,11], the impact of the use of pictures in web surveys [12-17], and advancements in technology enabling the use of pictures with ACASI mode [19-21], there is limited research on the use of pictures in the ACASI survey mode [8,18,22]. This study illustrated all the survey questions and response options with pictures and adapted an existing web-based ACASI application to administer a picture-assisted ACASI survey. The survey was created for the Myanmar-born community in Australia, who spoke different languages and had different English and Myanmar (Burmese) language competencies. The 5 sections included in survey were modes of hepatitis B transmission, hepatitis B blood tests, hepatitis B vaccination, sexual behaviors, and demographics. These domains of inquiry were considered sensitive topics for the target population (Myanmar-born community in Perth, Western Australia), given discrimination toward people living with or at risk of hepatitis B infection [24,25] and the socially censored nature of sexual behaviors in Asia [26].

### Objectives

This study describes the 2-phase process of developing a web-based ACASI application using illustrated pictures. Phase 1 involved the preparation of the ACASI elements, and phase 2 involved synchronizing all the elements into the web-based ACASI application and the adaptation of the application features. This study outlines the challenges, feasibility, and limitations of the development process and areas for future research.

## Methods

**Figure 1:**
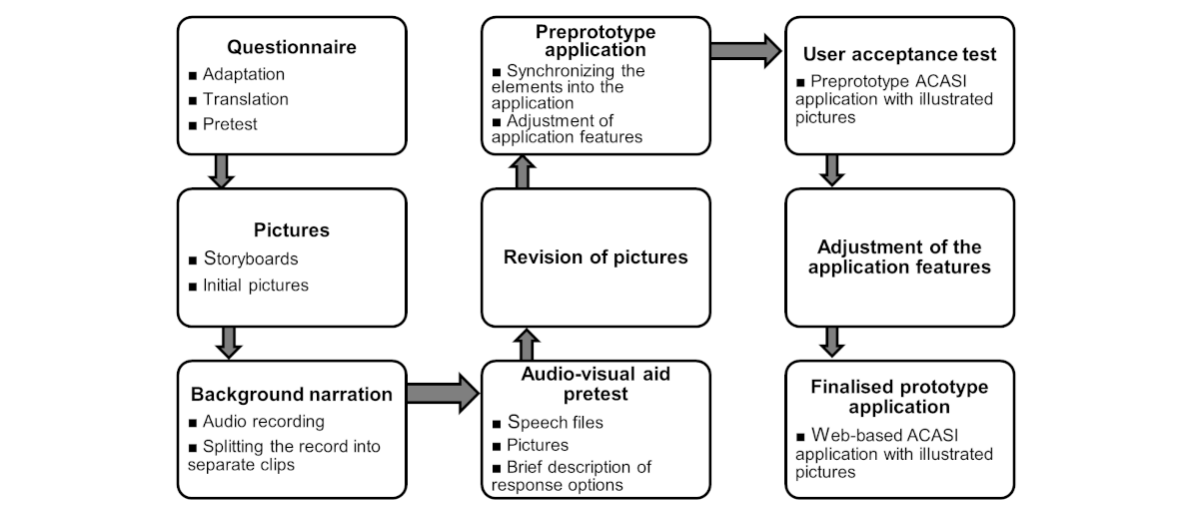
Development process of a web-based audio computer-assisted self-interview (ACASI) application with illustrated pictures.

The development of a web-based ACASI application with illustrated pictures in 2020 involved following two phases: (1) the development and pretesting of the elements of the application and (2) synchronizing the pretested elements into an existing ACASI application and the adaptation of the application features. The steps involved in application development are summarized in Figure 1.

### Phase 1: Development and Pretesting of the Elements of ACASI Application

Before the development of each element of ACASI application, potential application sources were explored. A range of ACASI application sources have been used in previous studies. Some researchers have purchased the subscription of ACASI platforms and built surveys on these platforms. Some studies contracted ACASI software developers who had adapted existing applications for each study. Other research teams have developed new applications that are specific to the corresponding studies. Examples of available ACASI platforms or software development services identified were Blaise, DatStat Illume Next, Questionnaire Development System, Research Triangle Institute, The Viewpoint Organization, Tufts University, and Westat [21,27-32]. A few other studies built study-specific ACASI software but did not report details about the sources of software [33-37]. Most ACASI applications are station based, that is, data are stored in the memory of the devices used, whereas a few applications are web-based and include cloud storage feature. In case of station-based data storage, there is a risk of loss of data if devices are lost [7]. The use of cloud storage feature requires a stable and secure internet connection.

Because internet access was readily available at the study location (Perth, Western Australia), a web-based ACASI application was selected for this study. The ACASI application was sourced from the Viewpoint Organization, which had developed web-based ACASI applications with different features customized to the aims of corresponding projects [32]. It was expected that adapting an existing ACASI application would save the time associated with overcoming the challenges of developing a new application. After identifying the application source, the elements of the ACASI application, namely, the questionnaire, pictures, and audio files, were developed and pretested.

The questionnaire topics included hepatitis B knowledge, attitudes, and practices. The questionnaire was adapted from similar previous questionnaires used among CaLD populations in Australia [38-41]. The questionnaire was developed in English using plain language and translatable word and verb forms [42]. Because the target population was the Myanmar-born community living in Perth, the questionnaire was translated into Myanmar language. The translation, review, adjudication, pretest, and documentation model [43] was used to guide this process. The translation, review, adjudication, pretest, and documentation model was applied such that the subject matter was translated into the target language in a contextualized and culturally appropriate manner [44]. Although several languages and dialects are spoken by the target community and we are aware of potential survey errors owing to limited proficiency of the national language among ethnic minority groups [45], because of research funding and time constraints, it was not practical to translate a questionnaire into several languages. Therefore, in this study, the questionnaire was made available in the national languages of the origin and receiving countries of the target community (Myanmar and English, respectively).

The questionnaire contained 40 questions. Only close-ended questions with multiple-choice response options were included. The forced answer option in the ACASI mode was used because respondents tend to skip questions unintentionally or intentionally, and the “forced answer” feature in web surveys has shown to overcome this issue [46,47]. To allow respondents to skip questions for ethical reasons, “I prefer not to answer” was included as a response option. The face validity of the English and Myanmar versions of the questionnaires was tested by 20 adults aged between 19 and 75 years (11 participants tested the English version and 9 tested the Myanmar version) from the target population, and the questionnaires were revised based on feedback. The face validity test participants were recruited from different areas of the Perth Metropolitan area and were of different ages, ethnicities, and educational backgrounds. It was time-consuming to develop, translate, pretest, and revise the questionnaire specific to this study. With the aim of developing an ACASI application, the use of a pretested questionnaire saved time for reprogramming the application at a later stage.

For the picture element of the ACASI application, the first author (NP) developed storyboards for each question and response item, because evidence suggests that specifications for the pictures should be determined by researchers, not by the artist [10]. Storyboards included specifications regarding numbers, features, manner, the facial expression of characters, and background supporting elements. A sample storyboard is presented in Table 1. The development of pictures included the consideration of many factors, such as consistency with textual information, cultural appropriateness, and simplicity. A review of the impact of pictures on health communication noted that pictures should be culturally relevant, and simple pictures were more useful than complex pictures [10]. Regarding the choice of characters, there is evidence that participants prefer characters that reflect their own appearance [48-51]. Because resources were not available to support the development of a customizable avatar feature, the characters for this study were designed to be gender and ethnicity neutral.

**Figure 2:**
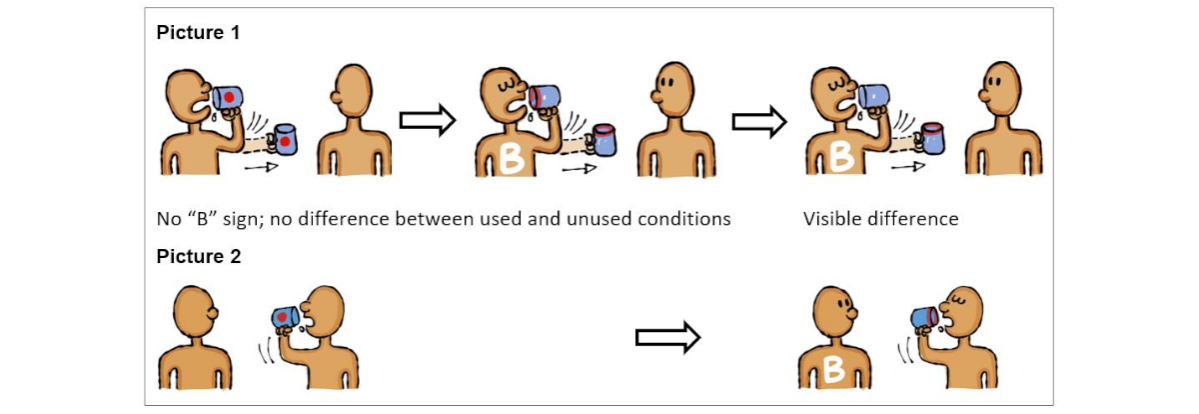
Development and revision of pictures based on the storyboard in Table 1.

The draft storyboards were reviewed, discussed, and revised by the research team. The questionnaire and revised storyboards were then presented to the artist who was based in Myanmar. The artist had 15 years of experience in creating comics, knowledge about hepatitis B, and an understanding of Myanmar’s culture. The artist used the Tayasui Sketch application [52] to develop and export pictures in JPEG format. Picture file sizes ranged from 31 KB to 441 KB. A series of revisions of the 2 pictures to illustrate the development of one question item is included in Figure 2. With regard to the accompanying text, a shortened form of each response option was agreed upon by the team because it was not possible to display the full response option text in the ACASI application. This meant the respondents would hear the full description in the audio while they would see a briefer description on the device screen, for example, “Could mean distrust,” was displayed instead of, “Suggesting to use condoms could mean distrust of a partner.”

At the same time as developing pictures, audio aids were developed. Text-to-speech (TTS) software was used to read the English questionnaire aloud in the ACASI application. For pretesting, the English questionnaire was read aloud using the Speak feature of Microsoft Word and was recorded using a voice recorder. The English voice file was approximately 12 minutes long. Owing to the high cost of the TTS software for the Myanmar language, a recorded speech was used for the Myanmar questionnaire. In reading aloud the questionnaire, to avoid measurement bias, the questionnaire was read aloud exactly in the way it was written [53]. An ACASI survey in Zambia reported that 15% of soundtracks are incorrect and 19% of soundtracks have poor sound quality because of background noise [37]. To enhance the quality of the speech file, a native Myanmar speaker read the questionnaire and recorded the voice in a studio. The Myanmar narration recording was approximately 15 minutes long. For time- and resource-efficiency, only one voice file was created using a woman’s voice. A woman’s voice was chosen based on evidence of better response rates and quality with woman interviewers in sexual and reproductive health surveys [54].

**Figure 3:**
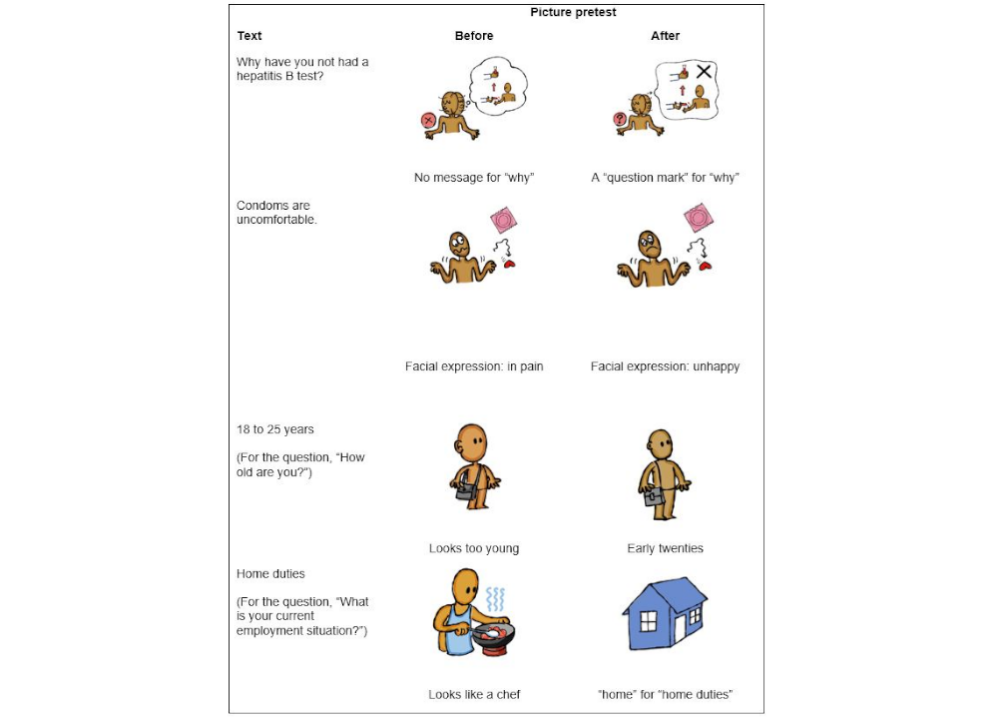
Revision of pictures after the pretest of pictures.

A pretest of audio-visual aids (audio narrations, pictures, and text to be displayed) was performed by the research team and the participants of 20 questionnaire face validity test. The draft pictures were reviewed for appropriateness and whether the pictures matched the corresponding text because viewers might interpret the same picture differently [10]. The shortened text to be displayed with each response option was also reviewed to assess whether the meaning was the same as the full text. On the basis of the review findings, the research team agreed on the pictures to be used in the application and those to be revised. After the revision of the 31 pictures, there were 138 pictures ready for use in the application. Most pictures were used only once, whereas a few were used repeatedly. The revision of the 4 pictures after the visual aid review is included in Figure 3. Voice recordings were reviewed for clarity, speech rate, and the preferred gender of the narrator. After reviewing the audio aids used in the ACASI application, the soundtrack of the Myanmar speech file was split into segments for each question and response option. In total, there were 210 audio segments. The segments were in the MP3 format.

**Table 1:** Storyboard for the development of 2 pictures to illustrate a question item.

Item			Description
Question text			Can hepatitis B transmission occur through sharing cups and spoons with a person who has hepatitis B?
**Pictures 1 and 2**			
	Characters	Two persons Person A: the person with hepatitis B infection Person B: the person without hepatitis B infection	
	Features of characters	Person A: a stickperson with a “B” sign (B in both languages if spaces allow) Person B: a stickperson (plain figure) Notes: half-body view	
	Facial expression of characters	Neutral	
**Picture 1**			
	Manner of characters	Person A: has a drink from a mug (new mug); *in another color or dotted line*, hands out the mug (stained mug) to Person B Person B: just standing with face toward Person A	
	Background supporting elements	A mug: no stain when drunk by Person A, visible stain when handed over to Person B (to avoid yellow color stain)	
	Image	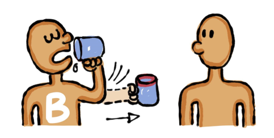	
**Picture 2**			
	Manner of characters	Person A: just standing with face toward Person B Person B: has a drink from the stained mug	
	Background supporting elements	A mug: visible stain when drunk by Person B (to avoid yellow color stain)	
	Image	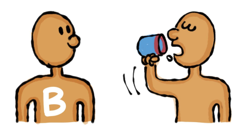	

### Phase 2: Synchronizing the Elements in the Web-Based ACASI Application and Adjusting the Application Features

The research team provided software programmers with the questionnaire, pictures, texts to display, wireframes (a sketch of each user interface), preferred color scheme, script for English narration, split speech files of Myanmar narration, and question skipping patterns. The research team designed only the user interface and not the administrator interface. In the user interface for survey respondents, ≥1 question items could be displayed on each page. In a study comparing scrolling (>1 question items per page) and paging (1 question item per page) designs of the same survey, a higher proportion of respondents left the survey on the first page with 8 question items than with 1 question item [55]. Therefore, only 1 question item was displayed on each page of the current application. There were 40 pages for 40 question items and 8 pages for welcome, consent, a page for each of the titles of 5 domains, and closing.

Each page could be locked as a portrait or landscape display or the “autorotate” feature could be enabled. Studies that compared the users’ perspective of portrait or landscape view on different devices for different tasks concluded that the portrait view was more commonly used than the landscape view, especially when apps were used on small mobile devices [56-58]. In the current application, because the aim was to use a device with a 25.7-cm screen display, the portrait view was chosen.

**Figure 4:**
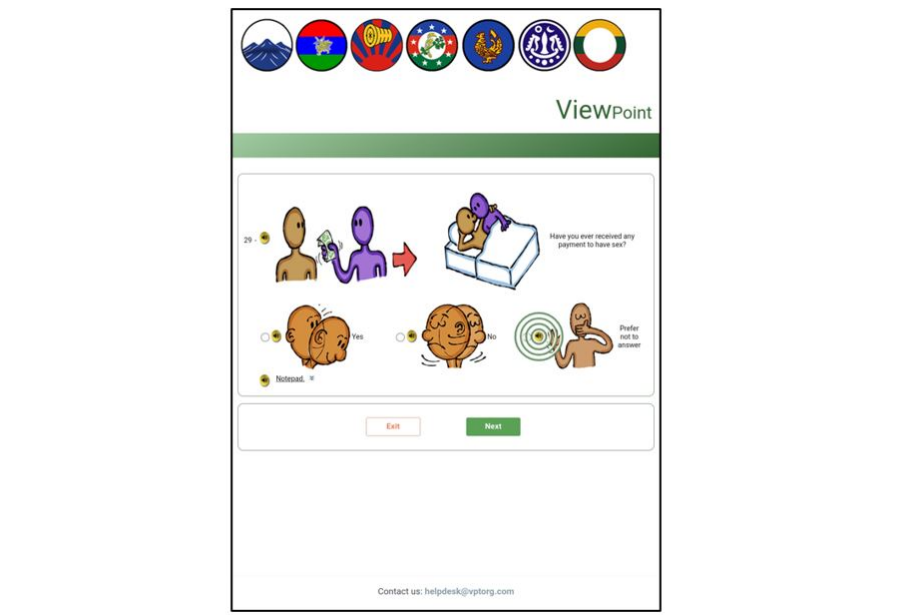
Speaker buttons with 2 functions in the web-based audio computer-assisted self-interview (ACASI) application with illustrated pictures.

Each page contained display-only texts, images, and interactive buttons. The display-only images were the logo of software developers, flags of the major ethnic groups of the target population, and pictures illustrating the questions (Figure 4). The flags were displayed to express the recognition of different Myanmar ethnic groups because the questionnaire was not translated into the multiple languages and dialects used in Myanmar. The display-only texts were messages on welcome, consent, closing, domain titles, and questions. The functional buttons were speaker buttons, radio buttons for response options (buttons, pictures, and text), and navigation buttons for “next” and “exit.” There was a push button in the footer space for survey respondents to seek help.

The audio component of the current application could be played automatically and on demand. Once each page was launched, the audio played automatically, and pulsing radio waves highlighted the speaker button next to the picture related to the corresponding audio narration so that respondents would be able to match what they heard to what they saw (Figure 4). When a user wanted to listen to a particular segment of the audio narration, the corresponding speaker button could be pressed. With the TTS function used for the English questionnaire, because the audio narration and text to be displayed for response options were different, the ACASI application was programmed with 2 lines of text, the displayed text and phonetic text, and the TTS function read the phonetic text aloud. The recorded speech files were used for the Myanmar questionnaire and stored on the server.

Regarding the position of pictures, a few studies examined the “banner blindness effect,” positioning the pictures or symbols before or after or left or right to the text, and the proximity of graphics to the text; however, there was no definitive evidence for best practice [12,14,59,60]. In the current application, the pictures were positioned before and left to the texts because they were used to convey the message, and it was intended that the users would look at the pictures while listening to the audio narration.

**Figure 5:**
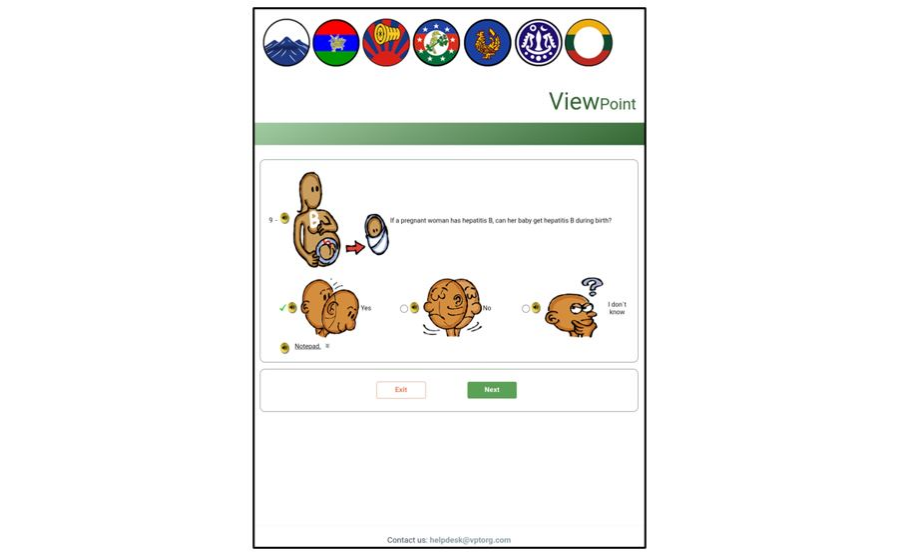
Changes in radio button styles in the web-based audio computer-assisted self-interview (ACASI) application with illustrated pictures.

Next to each response option picture were interactive radio buttons. The radio buttons were placed on the left-hand side of pictures and text because available literature suggests that this position could assist respondents in selecting the response options better than placing radio buttons on the right-hand side of the picture [61]. An empty circle was shown when a response option was not chosen, and a green check mark was shown after the response option was chosen (Figure 5).

**Figure 6:**
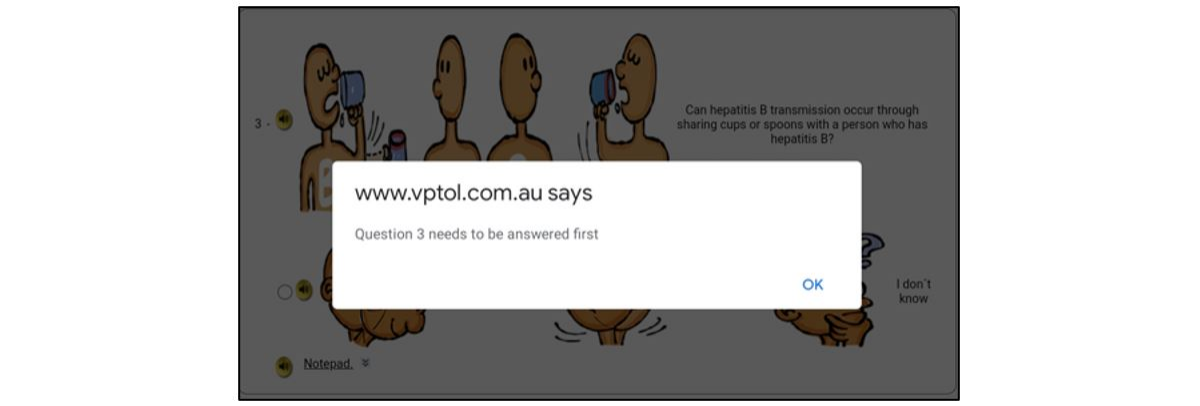
Prompts of the forced answer feature in the web-based audio computer-assisted self-interview (ACASI) application with illustrated pictures.

The green check sign assured the users that the selection had been made. In the current application, the forced answer feature is deployed because 2 previous experimental studies concluded that there was an association between a forced answer feature and low item nonresponse rates [46,47]. When a respondent pressed the “next” button without choosing any response options, an alert box popped up and reminded them to choose a response option (Figure 6).

Navigation buttons were included immediately below response options. In the current application, only the “next” navigation button was included, not the “back” button, and manual forwarding was applied. Automatic forwarding saves time because users are not required to select “next” for each question; however, previous studies have indicated that this could be associated with a high item nonresponse rate [62]. Automatic forwarding could be conditioned such that the page switches only after a response is entered. However, when the page did not switch unless the answer was entered, confusion was introduced, and consequently, high dropout rates or incompletion rates were observed [62]. Hence, manual forwarding with a forced answer feature was used in the current application, which required users to select the “next” button to progress to the next page. A “back” button was not included in the current application because there was no difference in item nonresponse rates or internal consistency in terms of whether the “back” button was included or not [63]. The browser back button was not disabled, so that previous pages could be accessed when required. On every page, there was an “exit” button that allowed users to quit the survey at any time.

Another study compared the positions of navigation buttons [64]. The study compared the position of the “next” button on the left-hand side or the right-hand side of the “back” button, and whether the 2 buttons should be close to each other. The findings indicated that participants preferred to see the “next” button on the right-hand side of the “back” button and to have the 2 navigation buttons close to each other. The layouts of the navigation buttons—“fixed at the top,” “fixed at the bottom,” and “right below the content of each page”—were compared by a further study among 62 participants [65]. This study concluded that data quality and respondent satisfaction were higher with the “right below the content” layout [65]. In the current application, immediately below the response options of each page, the “next” button was placed on the right-hand side of the “exit” button, and the 2 buttons were positioned close to each other.

The current application avoided the color yellow. A few qualitative studies have reported that jaundice or yellowish discoloration of the skin and sclera is a well-known symptom related to hepatitis B among migrants in Australia, and some misunderstood that all people living with hepatitis B had jaundice or those without jaundice did not have hepatitis B [66-69]. A white-colored background was used in the current application to ensure the clarity of the illustrated pictures.

Programmers synchronized the provided elements into the existing web-based ACASI application and adjusted the application features. The two new features added were as follows: (1) automatically played audio narration and (2) pictures hardwired to the response option buttons. In previous ACASI applications, speech played when the speaker button was pressed. In the adapted version, speech played automatically. In the current version, the pictures were included for both questions and response options, and pictures illustrating response options were hardwired to radio buttons. Among the existing features, the question skip feature was used in this study. The logic check feature was not included in this study because previous reports have suggested that it might take longer for the participants to go back to previous questions, introduce confusion, and interfere with the sense of privacy [8]. If the logic check feature was deployed, it would require “back” buttons.

The web-based ACASI application was written in HTML revision 5. The application was written to be used with any browser and to work on any computerized device and most mobile devices that had built-in sound and speakers. The base questionnaire was created in English from which a translation template was created. In the translation template, the questions and response options were translated into the Myanmar language, and then the Myanmar template was imported into the database.

The questions, responses, and pictures were built into a questionnaire database that allowed the analysis tool to monitor the progress of data collection, analysis, and export. The data were stored in the Structured Query Language database. The 2 servers used by ACASI application were a live production server used for production and a live spare server that mirrored the production server, which is available as an immediate backup. The ACASI application stored the data immediately after the response was entered. A respondent could not move on to the next page before the response was securely stored in the server. No data were stored in the device used; therefore, no storage space was used in the corresponding devices, and there was no risk of losing data if the device was damaged or stolen.

The application stored data in the English language, regardless of the language used by the respondents when completing the survey. The data were exported using the data extraction feature in Excel format, which could be configured for statistical software. Reports could be generated to show the languages used by the respondents. In addition, individual records could be displayed in the input language, although the data were stored in English language. The data analysis feature was for exporting individual records, frequency reports, cross-tabulation reports, and any open text.

### User Acceptance Test of the Preprototype Application

The research team and 5 participants from the target community conducted user acceptance testing of the preprototype ACASI application. The research team tested compatibility, functionality, and usability [70-72]. For browser and device compatibility, the application was first tested on an Android tablet intended for data collection and then tested on a Windows device and an iOS device. It was found that the application site had to be trusted to enable the autoplay feature of audio narration, and it worked best with the Chrome browser on Android and Windows devices. The audio was played only on demand on the iOS device. The application interface was larger than the display of the Android tablet. For functionality testing, the research team conducted 10 test surveys and tested the data extraction and analysis features to export and analyze the survey data. It was found that both data input and output features were functional. For usability testing, the research team checked whether all radio buttons, speaker buttons, navigation buttons, and push buttons were usable as programmed. The buttons were found to be usable, apart from the requirement to drag the pages with 4 or 5 response options to see the buttons of the last 1 or 2 options. Performance testing was not conducted because face-to-face recruitment was planned, the maximum volume would be only 3 participants at a time, and no web overload was expected. For site security, the research team tested the creation of user account, passwords, and logging in using the created credentials. Security was ensured by software programmers. Programmers also checked for the presence of defects or bugs.

The test users performed general user interface testing. They provided feedback on whether it was easy to use the application, hear and follow the speech, select and input their answers, and navigate to the next pages. To avoid the fatigue of pretest participants, it was decided not to seek detailed feedback on subjective user satisfaction regarding the general appearance of application interfaces, such as colors, font size and picture size, the functionality of speaker buttons and push buttons, and the clarity of instructions. After the review by the test users, suggestions for minimal changes were made. The test users commented that it was not user-friendly when they had to drag the page left and right to see all the contents on the touchscreen device. For pages with 4 or 5 response options in a row, the interface was larger than the frame of the device used during the user acceptance test; hence, not all the contents were visible without scrolling or dragging the page. Another comment was that when there were 4 or 5 response options in the same row and they chose the last or second last option, because those 2 options were not visible without dragging the page, they were confused whether their answer of choice had correctly been chosen. As a result, minimal adjustments were made to the preprototype application. The application interface was resized to autofit with the frames of the devices such that no scrolling or dragging would be required. The layout of the response options was limited to 3 in a row.

### Ethics Approval

This study was approved by the Curtin University Human Research Ethics Committee (HRE2020-034). The draft questionnaire and audio-visual aids were pretested by 20 adults recruited from the Myanmar-born community in Perth. In total, 5 Myanmar-born adults participated in a user acceptance test of the preprototype application. Informed consent was obtained from all the participants before data collection. No identifiable information was collected. For each pretest, each participant received Aus $20 (US $15.2) as an acknowledgment for their time. The pretest audio recordings and databases were stored in a secure storage facility at Curtin University.

## Results

After 1 year of preparation and development of the application, the prototype application was functional with ACASI survey features, such as audio narration, computerized survey administration, and data storage and export. Users were required to log in to the ACASI application with user-specific log-in credentials. The language choice option was displayed on the log-in and welcome page. The welcome, consent, title of each session, each question item, and closing page were shown on separate screens. For the 40 question items in the questionnaire of this study, the survey was shown on 48 separate pages in the application. It took 8-15 minutes to complete the survey, depending on the language used and whether the users selected responses before or after listening to the complete speech file of each page. The user had to press the “next” button to progress to the next page. There was an “exit” button on every page, which allowed users to quit the survey at any time. The narration played automatically once the user accessed each page. Touchscreen devices were used by respondents to enter the survey responses. To enter a response, the users had to tap on the corresponding picture. Because it did not require the users to press exactly on the circular radio button for each response option, it was easy for the users to enter their responses. When the user pressed “next” without choosing any response option, there was a prompt (Figure 6) saying, “Question number ‘n’ needs to be answered first.” The built-in question skip command assured that the “next” button navigated users to the next appropriate questions.

## Discussion

### Principal Findings

This paper describes the process of developing a picture-assisted ACASI application, including its challenges and good practices. In the first phase of the development process, the development and pretesting of questionnaires, pictures, audio recordings, and a brief description of the response options were presented. The second phase discussed the synchronization of pretested questionnaires and audio-visual aids into the existing web-based ACASI application; the technical information of the application; and the adaptation of the application features, such as the visual design of the user interface, the functions of radio buttons, navigation buttons, push buttons, and audio narration with pulsing radio waves, highlighting the corresponding response options.

Major challenges in the development of this web-based ACASI application with illustrated pictures were the time-consuming and resource-intensive nature of developing, pretesting, and revising pictures; splitting the audio files into separate tracks; setting up the questionnaire in the ACASI application; and adjusting the features of the existing web-based application. This finding is consistent with the time-consuming nature of designing, setting up, and implementing ACASI software reported by a study that piloted a station-based ACASI application in 3 languages in South Africa in 2008 [73].

The development process for pictures and audio files in this study was found to be useful because no major revisions were required. In this study, the artist had a cultural background similar to that of the target population. An ACASI study in India in 2010 required making pictures developed in the United States more discreet and culturally appropriate for study participants in India, and this warranted the reproduction of pictures by a local artist [74,75]. For the Myanmar-language questionnaire in this study, audio narration was recorded in a recording studio after pretesting and revision of the questionnaire. Kane et al [37], who developed a station-based ACASI application in 2 languages in 2013, made similar recommendations that questionnaires should be pretested before audio recordings, narrators should read aloud the script word-for-word, and the audio recordings should be free from background noise [37].

A good practice learned in this study is that the elements of ACASI were pretested and revised before being synchronized into the application. In doing so, time and other resources were saved to avoid making multiple changes during development and in a later version of the application. If changes were required, a new questionnaire database would be required rather than amending the former database, which would require additional time investment and associated costs. Similar recommendations have been made by researchers who formerly developed ACASI applications that translated questionnaires, pictures, and audio files should be pretested [7,37,74]. Pretesting each element of ACASI before the user acceptance test enabled the pretest participants to focus on each element and provide valuable feedback at each step.

### Limitations

The limitations of this study include the small sample size for the user acceptance test. However, the feedback collected from the user acceptance test participants was constructive in refining the preprototype version of the application. To improve the application further, an alert prompt can be accompanied by an audio track. The response options for radio buttons can be made larger. Each response option (radio button and picture) can be enclosed in a boundary that functions as a wide button. The speaker button can be enlarged and positioned on the left side of the corresponding wide button.

This study shares the experience of developing a web-based ACASI application with illustrated pictures. The newly developed application was found to be fully functional with essential ACASI web survey features, such as background audio narration, computer-assisted self-administration of surveys, question skip commands, secure data storage, and versatile data export. The new features added to the current application were illustrated pictures and autoplay audio features with pulsing radio waves. Including several elements in the web-based ACASI application requires careful consideration of multiple factors in its development and use. Pretesting each element was found to be a useful approach. On the basis of our experiences, we offer suggestions to assist other researchers during phase one of the development of a web-based ACASI application with illustrated pictures.

1. Agree on the specifications of each element of ACASI at the beginning of the process: The type of ACASI application to be used must be determined at the beginning of the process such that application-specific picture file formats can be produced. For example, 4 classes of bitmap file formats were required for each picture button in Questionnaire Development System ACASI software [76].
2. Shorten the preparatory phase: It would save time to use a pretested questionnaire in the development of a similar ACASI application in the future. A shared repository of pictures can be developed from which pictures can be reused whenever relevant.
3. Itemize audio tracks: When the background narration has to be recorded in multiple languages and if the person responsible for splitting the audio files into separate tracks does not understand the languages, each separate track can be numbered, and the narrator can read the numbers in English during recording.
4. Consult consumers on pictures and application interface: If time and resources allow, representatives of the target population should be consulted for picture development and visual design of the application.
5. Pretest each element before developing an ACASI application or setting up the elements on an ACASI platform: This would save time and resources to reprogram the application at a late stage.

With the possibility of using pictures in ACASI applications, ACASI with pictures could be further tested as an innovative mode of administering surveys about sensitive topics among people who use different languages and dialects, who have different competencies of the languages used in the survey, and who have different literacy levels.

## Appendix

Multimedia Appendix 1 Audio computer-assisted self-interview (ACASI) application with illustrated pictures demonstration video.

## Abbreviations

ACASI: audio computer-assisted self-interview
CaLD: culturally and linguistically diverse
TTS: text-to-speech

## Data Availability

A screen capture of 1 question item in web-based ACASI application in this study with illustrated pictures can be found in Multimedia Appendix 1. The pretest data are available on request.
